# Analysis of Quorum-Sensing Pantoea stewartii Strain M073a through Whole-Genome Sequencing

**DOI:** 10.1128/genomeA.00022-15

**Published:** 2015-02-19

**Authors:** Nur Izzati Mohamad, Wen-Si Tan, Chien-Yi Chang, Kok Keng Tee, Wai-Fong Yin, Kok-Gan Chan

**Affiliations:** a Division of Genetics and Molecular Biology, Institute of Biological Sciences, Faculty of Science, University of Malaya, Kuala Lumpur, Malaysia; b Interdisciplinary Computing and Complex BioSystems (ICOS) Research Group, School of Computing Science, Newcastle University, Claremont, Tower, Newcastle upon Tyne, United Kingdom; c Centre for Bacterial Cell Biology, Medical School, Newcastle University, Richardson Road, Newcastle upon Tyne, United Kingdom; d Department of Medicine, Faculty of Medicine, Centre of Excellence for Research in AIDS (CERiA), University of Malaya, Kuala Lumpur, Malaysia

## Abstract

Pantoea stewartii strain M073a is a Gram-negative bacterium isolated from a tropical waterfall. This strain exhibits quorum-sensing activity. Here, the assembly and annotation of its genome are presented.

## GENOME ANNOUNCEMENT

Isolated from a tropical waterfall in Sungai Tua located in the Selangor state of Malaysia, strain M073a belongs to the genus Pantoea stewartii, members of which have been reported as pathogens known to cause the Stewart’s wilt disease, which infects sweet corn (1). In young seedlings, Stewart’s wilt causes wilting, which usually result in death of the seedling. In mature plants, leaf blight can be seen parallel to the veins of the leaves. This blight later forms dark-colored necrotic lesions. In some hybrid varieties of sweet corn only small lesions are formed (1 to 2 cm), which is dependent on the level of resistance (2). Members of Pantoea are Gram-negative bacterium, and most of them show quorum-sensing (QS) activity (3–5). It has been reported that P. stewartii is endemic in various states of the United States (6). Causing disease that commonly occurs in the winter and late summer, Pantoea spp. can be found in various habitats from the western borders to the southern and northern coast, including the Plains (7). Here, we present the draft genome of Pantoea stewartii strain M073a for the better understanding of the relationship between its pathogenicity and its QS activity.

Genomic DNA of strain M073a was extracted using the QIAamp DNA minikit (Qiagen, Germany) following the instructions on the kit’s manual. Quality of the genomic DNA extracted was evaluated using the NanoDrop spectrophotometer (Thermo Scientific, Waltham, MA, USA) and further quantified with the Qubit 2.0 fluorometer (Life Technologies, Carlsbad, CA, USA). The whole genome of strain M073a was sequenced using an Illumina MiSeq sequencer and generated 1,509,201 filtered reads with 56-fold coverage. The reads were then assembled *de novo* using the CLC Genomic Workbench 5.1 (CLC Bio, Denmark). A total of 39 contigs were yielded, with an *N*_50_ value of 375,223. The resulting draft genome of P. stewartii strain M073a was 4,817,607 bp in length with 53% GC content. Prodigal 2.6 software was used for gene prediction and resulted in 4,298 open reading frames (ORF) (8). The Rapid Annotation using Subsystem Technology (RAST) server was used for the annotation of predicted genes (9) and tRNAscan-SE (version 1.21) (10) was used to predict the number of tRNAs, which was 68. RNA genes were predicted using the RNAmmer software (11), with which three copies of 5S rRNA genes, one copy of a 16S rRNA gene, and one copy of a 32S rRNA gene were found in this genome analysis.

From the annotated genes of the draft genome, there are two genes that play key roles in the QS mechanism, *luxI* and *luxR*, which are found in contig number 14. This finding is consistent with a study conducted by Tsai and Winans in 2010 which report the discovery of LuxI and LuxR homologues which are in the EsaI/EsaR system in P. stewartii (12). Since P. stewartii regulates its virulence via QS (1), our work on the whole-genome sequencing of P. stewartii strain M073a could serve as a QS model to study the QS relationships in this pathogen.

### Nucleotide sequence accession numbers.

This whole-genome shotgun project has been deposited at DDBJ/EMBL/GenBank under the accession number JSXF00000000. The version described in this paper is the first version, JSXF01000000.
